# Intergenic transcription in in vivo developed bovine oocytes and pre-implantation embryos

**DOI:** 10.21203/rs.3.rs-2934322/v1

**Published:** 2023-05-19

**Authors:** Saurav Ranjitkar, Mohammad Shiri, Jiangwen Sun, Xiuchun Tian

**Affiliations:** University of Connecticut; Old Dominion University; Old Dominion University; University of Connecticut

**Keywords:** Intergenic transcription, read-out, read-in, read-through (down-stream of gene)

## Abstract

**Background:**

Intergenic transcription, either failure to terminate at the transcription end site (TES), or transcription initiation at other intergenic regions, is present in cultured cells and enhanced in the presence of stressors such as viral infection. Transcription termination failure has not been characterized in natural biological samples such as pre-implantation embryos which express more than 10,000 genes and undergo drastic changes in DNA methylation.

**Results:**

Using Automatic Readthrough Transcription Detection (ARTDeco) and data of *in vivo* developed bovine oocytes and embryos, we found abundant intergenic transcripts that we termed as read-outs (transcribed from 5 to 15 kb after TES) and read-ins (transcribed 1 kb up-stream of reference genes, extending up to 15 kb up-stream). Read-throughs (continued transcription from TES of expressed reference genes, 4–15 kb in length), however, were much fewer. For example, the numbers of read-outs and read-ins ranged from 3,084 to 6,565 or 33.36–66.67% of expressed reference genes at different stages of embryo development. The less copious read-throughs were at an average of 10% and significantly correlated with reference gene expression *(P* < 0.05). Interestingly, intergenic transcription did not seem to be random because many intergenic transcripts (1,504 read-outs, 1,045 read-ins, and 1,021 read-throughs) were associated with common reference genes across all stages of pre-implantation development. Their expression also seemed to be regulated by developmental stages because many were differentially expressed (log_2_ fold change ≥ 2, *P* < 0.05). Additionally, while gradual but un-patterned decreases in DNA methylation densities 10 kb both up- and down-stream of the intergenic transcribed regions were observed, the correlation between intergenic transcription and DNA methylation was insignificant. Finally, transcription factor binding motifs and polyadenylation signals were found in 27.2% and 12.15% of intergenic transcripts, respectively, suggesting considerable novel transcription initiation and RNA processing.

**Conclusion:**

In summary, *in vivo* developed oocytes and pre-implantation embryos express large numbers of intergenic transcripts, which are not related to the overall DNA methylation profiles either up- or down-stream.

## Background

The mammalian genome consists of ~ 35% of introns and just 2.8% exons [1]. Recent studies showed, however, that more than 85% of the human genome is transcribed [2]. These data suggest that large portions of the intergenic regions of the genome are also transcribed. Indeed, failure of transcription termination and novel transcription initiation such as long non-coding RNAs (lncRNAs) and long intergenic non-coding RNA (lincRNA), some from gene-poor regions of the genome [3], have both been described, with the first category accounting for up to 20% of intergenic transcription [4]. Intergenic transcripts due to termination failure were present in cultured cells and enhanced in quantities and lengths under proteotoxic stress such as heat and osmotic pressure, as well as viral infection [5, 6]. The observations that such intergenic transcripts did not exit the nucleus, and their enhanced transcription was correlated with locus decompaction and binding of transcription factors to heterochromatin regions [4] suggest that intergenic transcripts may play a role in maintaining chromosomal integrity and euchromatin structures [7].

A number of software packages have been published to classify and quantify intergenic transcription near genes and possibly caused by termination failure. Automatic Readthrough Transcription Detection (ARTDeco) distinguishes itself by expanding prior approaches with the implementation of three categories of intergenic transcription, all closely associated with references genes. First, read-out transcription is detected 5 kb down-stream from the termination end site (TES) and 100 bp to 15 kb in length. Second, read-in transcription is detected 1 kb up-stream of transcription start site (TSS) and extends up to 15 kb in length. Finally, read-through (also known as down-stream of genes) is continuous transcription after TES and between 4 to15 kb in length [8]. These classifications separate bona fide transcription termination failure from potentially new intergenic transcription initiation near genes.

DNA methylation has long been known to regulate gene expression and chromatin organization [9]. While the hypomethylation of CpG islands in promoter regions of genes is an important mechanism for transcription and gene expression, hypermethylation of the gene body is positively correlated with gene expression [9, 10]. While methylation of intergenic regions has been hypothesized to also maintain genome stability [11], whether it play a role in intergenic transcription has not been investigated.

The presence of transcription factors binding motif is a mechanism for initiation of transcription intergenically. While transcription factor binding motifs [12, 13] and polyadenylation signals [14] have been identified in certain lncRNAs and lincRNAs, they have not been studied for intergenic transcripts that are caused from termination failure or those that are closely associated with genes [15, 16].

Intergenic transcription caused by failure of termination has also not been studied in natural biological samples. Bovine oocytes and pre-implantation embryos express on average of 10,000 genes and undergo dramatic methylation level and pattern changes across developmental stages [10, 17]. We set forth to determine 1) to what extent is intergenic transcription present in bovine pre-implantation development, 2) if a correlation exists between intergenic transcription and DNA methylation patterns, 3) if intergenic transcripts are preceded with transcription factor binding motifs, and 4) if intergenic transcripts contain polyadenylation sequences.

Here we document widespread intergenic transcription in matured oocytes and early embryos that were not correlated with DNA methylation. Transcription factor binding motifs and polyadenylation sequences were found in many intergenically transcribed regions suggesting new transcription initiation and transcript stabilization.

## Results

### Identification and Quantification of Read-outs, Read-ins, and Read-throughs

**Figure 1:**
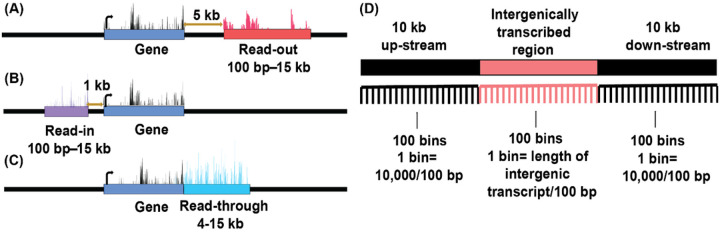
Graphical representation of the three categories of intergenic transcripts detected by ARTDeco and binning scheme for methylation analysis. (A) Read-outs (red): transcribed from 5–15 kb after TES of the reference gene (blue). (B) Read-ins (purple): transcribed 1 kb up-stream of the reference gene, extending up-stream up to 15 kb. (C) Read-throughs (cyan): continuous transcription from TES of the reference gene, 4–15 kb in length. (D) The 10 kb up- and down-stream (black) of and including the intergenically transcribed regions (pink), a 20 kb+ span of the genome was divided into a total of 300 bins. While the 10 kb up- and down-stream regions were equally binned (100 bins of 100 bp each), the bin size for intergenically transcribed regions were dependent on their lengths, with the 100 bp to 15 kb intergenically transcribed regions binned as 1 bp to 150 bp per bin.

**Figure 2:**
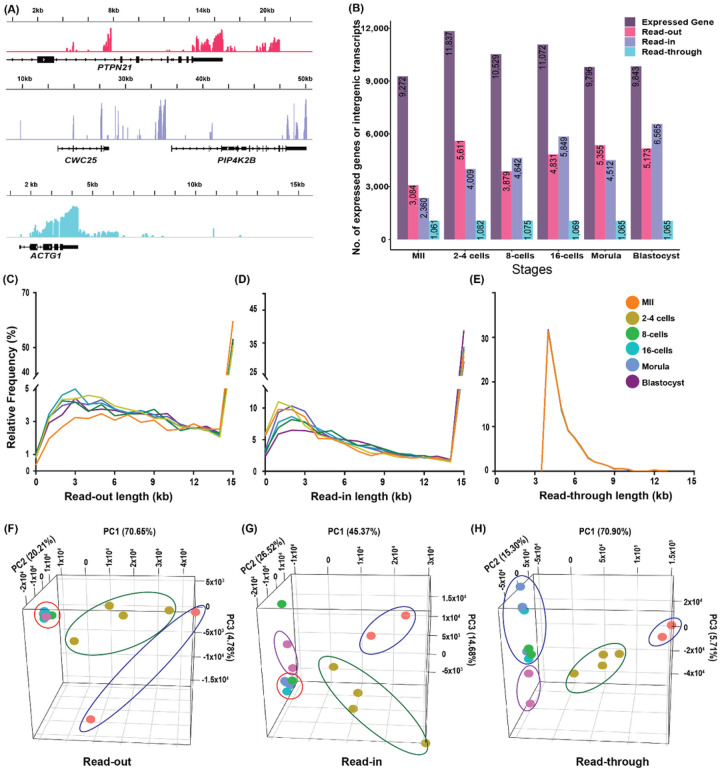
Abundance, lengths and principal component analyses (PCA) of intergenic transcription in oocytes and pre-implantation embryos detected by ARTDeco. (A) Representatives of a read-out, read-in, and read-through transcript found in a blastocyst. The horizonal lines represent the regions of the genome transcribed. The colored vertical bars are relative read counts. Gene names were given below the black vertical lines which represent exons, and the arrows show directions of transcription. The top panel (pink) shows read-out transcription of the *PTPN21* gene. The middle panel (purple) shows read-in transcription starting up-stream of the *PIP4k2B*gene. The bottom panel (teal) refers to read-through transcription generated from the continuous expression of the *ACTG1* gene. (B) The total numbers of expressed genes, read-outs, read-ins, and read-throughs detected at various stages of pre-implantation development. The densities of various lengths of read-outs (C), read-ins (D) and read-throughs (E) of all samples combined were also plotted. PCAs of read-outs (F), read-ins (G) and read-throughs (H) for various stages of pre-implantation development are shown in three dimensions. Color keys are the same as shown in (E). Dots in blue boundary are MII oocytes, in green are 2–4 cell embryos, in red are 8-cells to morula or blastocysts, and blastocyst are inside the purple boundary.

ARTDeco identified, located, and quantified all categories of intergenic transcription from RNA-seq data of pre-implantation development. ARTDeco also identified all expressed genes as references to locate intergenic transcripts. The numbers of expressed genes identified by ARTDeco were within the same range (~ 10,000 genes) as we reported previously [18]. To illustrate intergenic transcription, we plotted representatives of a read-out, read-in, and read-through transcripts (see definitions and Fig. 1 in Methods) of a blastocyst in Fig. 2A. The top (pink), middle (purple) and bottom (teal) panels show the read-out for the *PTPN21*gene, read-in for the *PIP4k2B* gene, and read-through for the *ACTG1* gene, respectively, all transcribed outside of the reference gene regions.

Figure 2B shows the numbers of each type of intergenic transcript as well as the numbers of the expressed genes. We revealed wide-spread intergenic transcription among different stages of pre-implantation development. For example, among the 9,796 genes expressed at the morula stage, 5,355 were found to have corresponding read-out transcripts, accounting for 54.67% of the expressed reference genes. Among different stages of development, the same high percentages of expressed genes, ranging from 33.26–54.67%, were associated with read-out transcripts. Similarly, 25.45 to 66.70% of expressed genes had read-in transcripts. The highest number of read-in transcripts, 6,565, was found at the blastocyst stage, accounting for 66.70% of the 9,843 expressed genes. Of note, MII stage seemed to be low in both read-outs and read-ins. The percentages of expressed genes associated with read-throughs, however, were much lower and less varied among different stages. The range was between 9.14% (2–4 cell stage) and 11.44% (MII). The abundance differences among different types of intergenic transcripts may be explained by the parameters used to identify them (Fig. 1A–C). Read-throughs were defined as 4–15 kb in lengths while read-outs and read-ins both included transcripts as short as 100 bp and as long as 15 kb.

To compare the overall expression levels of intergenic transcripts to those of expressed genes (TPM ≥ 1), we calculated the percentage of read counts of intergenic transcripts over those of expressed genes by developmental stages. The read counts of expressed intergenic transcripts were magnitudes lower than those of the expressed genes. For example, the highest percentages of total read counts for read-outs and read-throughs over those of all expressed genes (relative read counts) were 1.3% and 13.76% in MII stage, respectively (Supplementary Table 1A). For read-in, the relative read count was highest at the 2–4 cell stage (1.6%) (Supplementary Table 1A). Interestingly, there were ample numbers of intergenic transcripts that were associated with reference genes that were un-expressed (TPM < 1). Overall, these are 64.90% for read-outs, 53.54% for read-ins and 30.32% for read-throughs (Supplementary Table 1B). These observations suggest that one third to more than half of the intergenic transcripts detected by ARTDeco, are new initiation and not generated by termination failures.

We also analyzed the lengths and distribution of each type of intergenic transcripts. The majority of the read-outs and read-ins were between 2–3.5 kb in length (Fig. 2C, 2D). Transcripts of other lengths were minor in comparison, especially after 3.5 kb, suggesting that the RNA polymerases eventually fell off the chromatin. These length results corresponded with previous published data of cell culture, which showed that the number of intergenic transcripts were inversely proportional to their lengths [5, 6, 19]. Interestingly, we observed upticks in transcript abundance passed 15 kb for both read-ins and read-outs. This likely happened because ARTDeco combined all densities of read-ins and read-outs longer than 15 kb, and artificially inflated their numbers passed this milestone length. Similarly, the lengths of read-throughs ranged between 4–9 kb with the majority found at 4–5 kb (Fig. 2E). No uptick in read-through lengths was present because read-throughs were identified last in order to avoid double counting of transcription.

Interestingly, no prior studies on intergenic transcription conducted PCAs, likely because of the assumption that intergenic transcription could be random in nature. Yet PCA here revealed prominent segmentation by embryonic stages in all categories of intergenic transcripts. Separation was seen in three clusters for read-outs: MII oocytes, 2–4 cells, and all stages post-EGA (Fig. 2F). For read-ins and read-throughs, four clusters were seen. While oocytes and 2–4 cell stages had their own clusters as in read-outs, blastocysts segregated from the other post-EGA stages (Fig. 2G, 2H). These patterns showed that similar to changes in gene expression [18, 20], intergenic expression also changed as pre-implantation development progressed, and major events such as EGA and blastulation played important roles in their expression regulations.

### Chromosome- and Stage-Specific Patterns of Intergenic Expression

**Figure 3:**
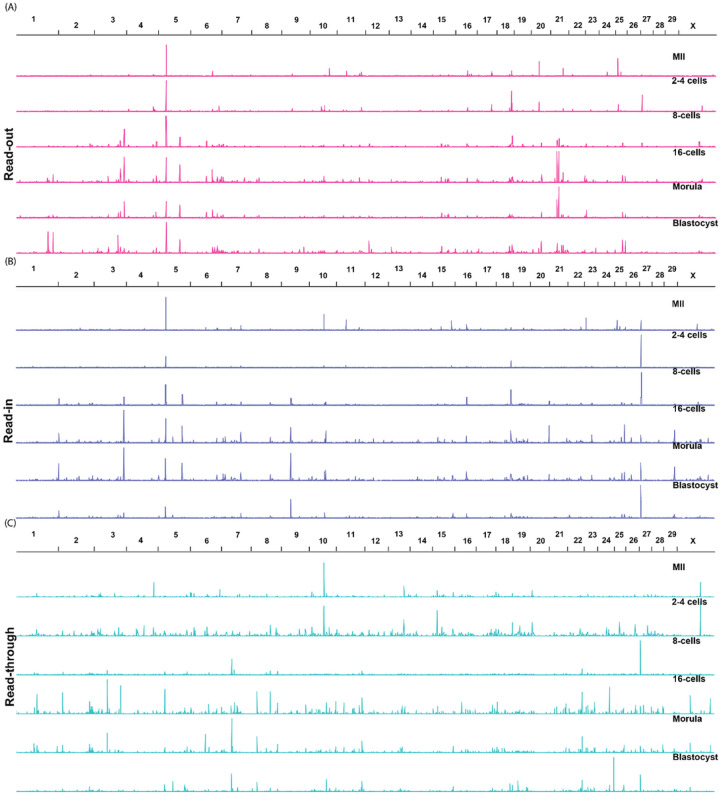
Visualization of intergenic transcription patterns by chromosome (horizontal black lines with numbers). The locations (colored horizonal lines) and relative read counts (colored vertical bars) for read-outs (A, pink), read-ins (B, purple) and read-throughs (C, teal) of all developmental stages were plotted for each chromosome. All intergenic transcripts were expressed with TPM≥1.

To analyze if there were chromosomal patterns of intergenic transcription, we plotted normalized bigwig files for all stages of development (Fig. 3). There appeared to be “hot spots” of intergenic transcription among all stages studied. A good example could be seen for read-outs on Chromosome 5 (Fig. 3A). Distinct intergenic transcription for read-out before and after EGA was also represented on Chromosomes 3. The averaged TPM of read-out transcription on this chromosome before and after EGA were 36.26 and 240.24, respectively (Supplementary Table 2A), and for Chromosome 21 they were 58.31 and 181.97, respectively (Supplementary Table 2A). These data therefore supported the visual patterns. Similar patterns could also be identified for read-in transcription such as those in Chromosomes 12 (before EGA and after EGA: 26.7 and 187.97, respectively, Fig. 3B, Supplementary Table 2B). While the transcription patterns for read-throughs were less obvious across stages, regions of enhanced read-through transcription were clearly present before EGA and after EGA such as those seen on Chromosome 10 (before EGA and after EGA: 1084.65 and 709.46, respectively; Fig. 3C, Supplementary Table 2C) and on the X chromosome (before EGA and after EGA: 1976.83 and 970.66, respectively; Supplementary Table 2C). Different overall intergenic transcription patterns were also seen along most chromosomes between the 16-cell and morula stages.

Interestingly, not only were there “hot spots”, each type of intergenic transcription seemed to have its own low activity developmental stages. Examples of these were 2–4 cell stage for read-outs (averaged TPM for the stage: 194.16), and blastocyst stage for both read-ins (averaged TPM for the stage: 186.20) and read-throughs (averaged TPM: 887.33) (Supplementary Table 2A–C). Additionally, within each development stage, chromosomes also varied in their intergenic activities. For example, highly active chromosomes for read-outs, read-ins and read-throughs seemed to be 20 (averaged TPM: 1372.63), 22 (averaged TPM: 1198.41) and 28 (averaged TPM: 1916.69), respectively, based on the averaged TPMs for all stages. Similarly, less active chromosomes for intergenic transcripts were 12 for read-out, 9 for read-in, and 29 for read-through (see Supplementary Table 2A–C for averaged TPM values). Taken together, the gross visible patterns and averaged TPM values by developmental stage and/or chromosome confirmed the results from PCA that intergenic transcription was not random. Rather, it was either related to the expression of their reference genes or subjected to specific regulations, possibly by genome sequence features or epigenetic markers.

**Figure 4:**
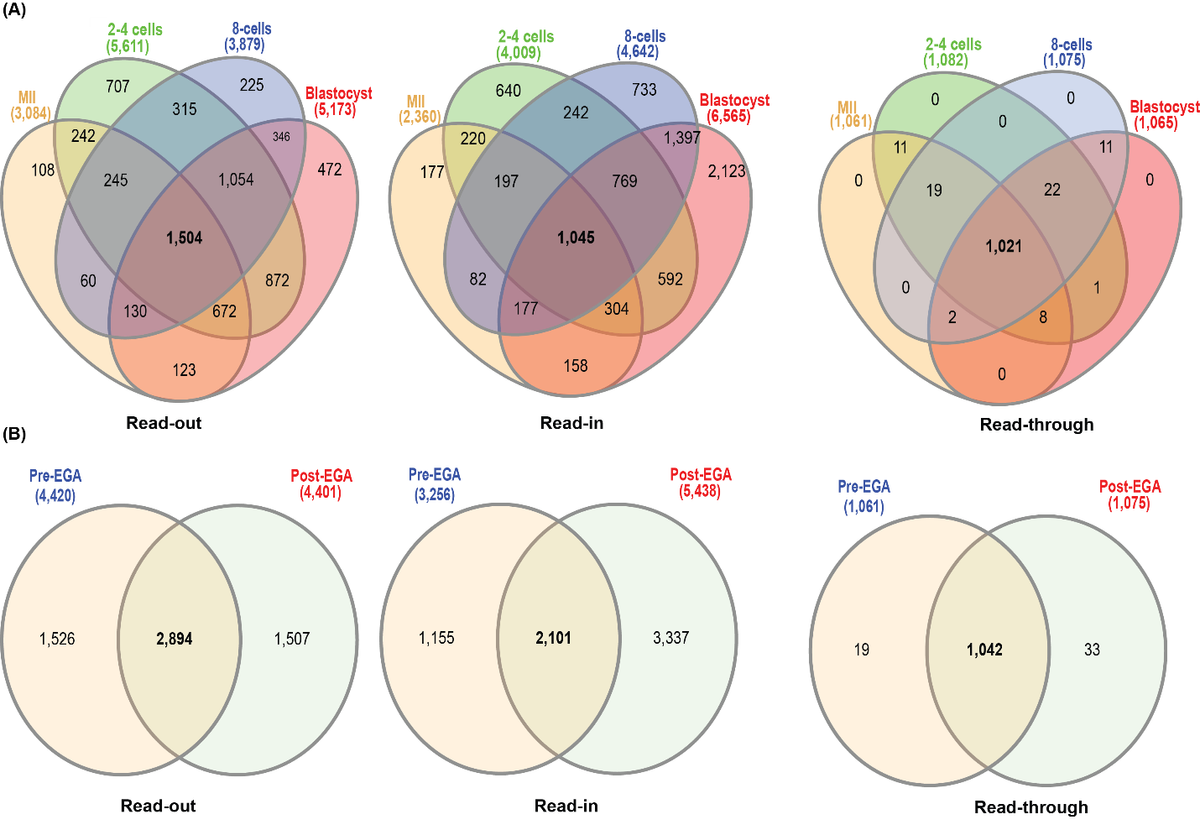
Comparative analysis of intergenic transcription in pre-implantation development. (A) Venn diagrams showing the identification of 1,504, 1,045 and 1,021 common reference genes for read-outs (left panel), read-in (middle panel), and read-throughs (right panel), respectively, in MII, 2–4 cells, 8-cells and blastocysts. (B). Venn diagrams showing common reference genes for read-outs (left), read-ins (middle) or read-throughs (right) between pre- (MII, 2–4 cell) and post-EGA stages (8-cell to blastocyst).

To identify the specific common regions of the genome that produced the intergenic transcripts, we conducted Venn analysis and found 1,504, 1,045 and 1,021 common reference genes that were associated with read-outs, read-ins and read-throughs, respectively, among all stages of pre-implantation development (Fig. 4A). The identification of high proportion of common reference genes for read-throughs was surprising at first but could be a consequence of the size of read-throughs (> 4 kb), which might have skewed their identification to the common and highly expressed ones. The fact that a low proportion of reference genes for read-outs and read-ins were common among different stages suggests that the majority of these transcripts may be stage-specific.

We then tried to determine the functions of the common reference genes. Reference genes for read-outs and read-ins had 11 and 34 GO terms, respectively (Supplementary Table 3). For read-outs, the major GO term was snRNA binding whereas common reference genes for read-ins were more involved in protein binding (Supplementary Table 3A–B). The GO terms for common reference genes of read-throughs, however, were represented by fewer transcripts per term and therefore a high number of GO categories, 414 in total. These also included protein binding as the top molecular function (Supplementary Table 3C).

We then analyzed the common intergenic transcripts before and after EGA. We observed 2,894, 2,101 and 1,042 common reference genes of read-outs, read-ins and read-throughs, respectively (Fig. 4B). These observations again suggest that intergenic transcription was not random.

### Differential Intergenic Expression Across Stages of Development

While many common read-outs, read-ins and read-throughs were found among different stages of development, numerous common transcripts were differentially expressed between stages (Table 1). The biggest difference appeared to be between 2–4 cells vs. blastocysts. For example, as many as 551 read-outs were differentially expressed between these stages. The highest and lowest differential read-ins were detected while comparing 2–4 cells and blastocyst (937), and 8-cells and blastocyst (5), respectively. For read-throughs, as many as 530 differentials were found between MII oocytes and blastocysts (Table 1).

### Expression Correlation of Intergenic Transcripts to Reference Genes

**Figure 5:**
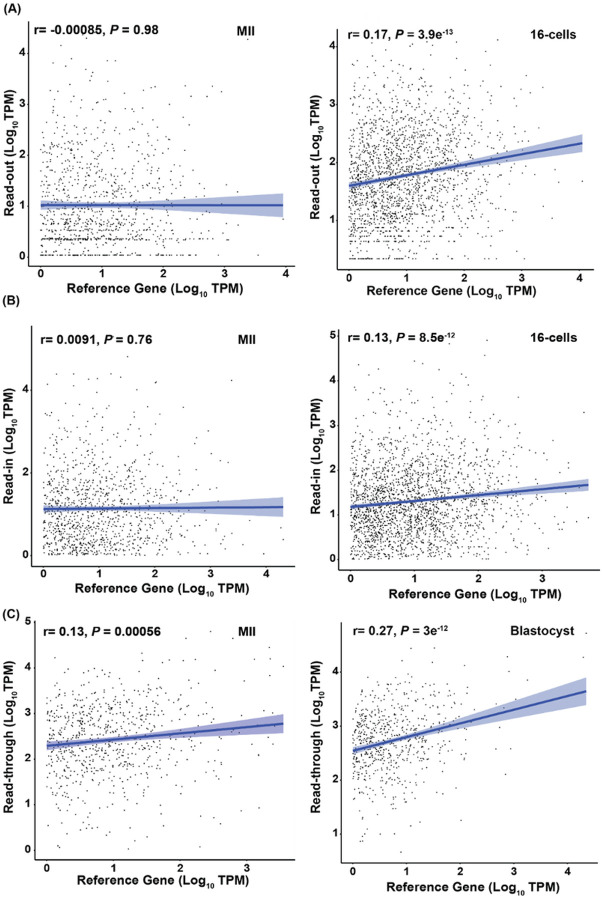
Pearson’s correlations between the levels of intergenic transcripts and those of their reference genes. Only the highest and lowest correlations among all stages for each type of intergenic transcription are presented here and the rest are in Supplementary Figure S1. The TPM values for read-outs, read-ins, read-throughs and their reference genes were transformed to the log_10_ scale before analysis. Each dot in the plot represents the log-transformed TPM value for a read-out (A), read-in (B) or read-through (C) against their expressed reference gene.

While our findings suggested a lower global intergenic transcription than the expressed genes, we found low to moderate correlation between the levels of expression of specific intergenic transcripts and their reference genes. Read-out transcription showed the highest correlation at the 8-cell (r = 0.15, *P* = 1.3e^− 08^) and 16-cell (r = 0.17, *P* = 3.9e^− 13^) stages (Fig. 5A, Supplementary Figure S1A). While significant, the r values were low and therefore not correlated. All other stages did not have any significant correlations (Supplementary Figure S1A). The patterns and degrees of correlation were similar in read-ins with the highest r values also found at the 8 cell (r = 0.12, *P* = 4e^− 08^) and 16-cell (r = 0.13, *P* = 8.5e^− 12^) stages (Fig. 5B, Supplementary Figure S1B). The only other stage that had a significant correlation was blastocyst (r = 0.1, *P* = 3.6e^− 07^; Supplementary Figure S1B). This was consistent with the fact that the transcription for read-outs and read-ins are not regulated by promoters of their reference genes. Higher r values, however, were found for read-throughs and their reference gene expression. Blastocyst stage had a r value of 0.27 (*P* = 3e^− 12^) and morula had the lowest yet significant correlation of r = 0.13 (*P* = 0.001; Fig. 5C, Supplementary Figure S1C).

### Methylation Profiles Encompassing Intergenically Transcribed Regions

**Figure 6:**
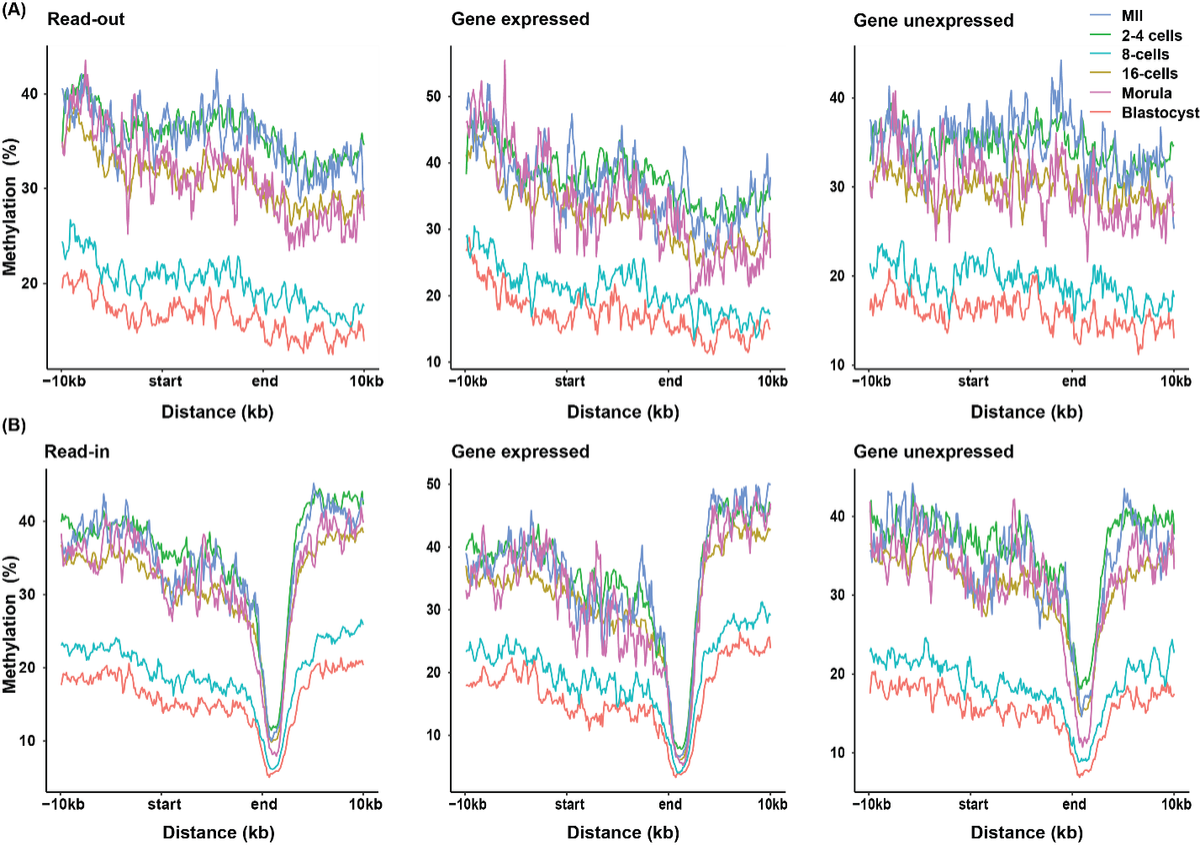
DNA methylation density plots for genome regions up- and down-stream of the read-out (A) and read-in (B) transcriptions for all stages of embryo development. The start and end are the beginning and end of the intergenic transcripts. Methylation density was investigated for all intergenic transcripts (left panels), intergenic transcripts associated with expressed (middle panels; TPM≥1) or unexpressed reference genes (right panels, TPM<1). The methylation analysis for read-throughs was highly variable and did not have any gradual decreasing patterns or low methylation at the 8-cell or blastocyst stages (Supplementary Figure S2).

The overall lack of correlations between intergenic transcripts and their reference genes suggests that specific regulations may be present for intergenic transcriptions. We therefore determined if methylation of the intergenically transcribed chromosomal regions was correlated with the level of transcription. We first screened 10 kb up- and down-stream of each transcribed read-outs, read-ins and read-throughs for overall methylation pattern visualization. We found a gradual decrease in methylation levels up-stream of read-outs (Figure 6A, left panel). A mild but noticeable increase in methylation levels was also noticeable throughout the bodies of the read-out transcripts. This mild increase dissipated after the end of the read-out transcription. These methylation patterns mimicked the much more pronounced patterns we reported for expressed genes in early embryos, i.e., low methylation at promoters and high methylation along the gene body and low methylation again past TES [10]. Also similar to methylation of genes in early embryos, we found lower levels of methylation along the entire 20 kb+ span including the intergenically transcribed regions in 8-cell and blastocyst stage embryos. Yet, the overall lowly methylated developmental stages did not generate more gene expression [10] or read-out expression. Together, these observations suggest that intergenic transcription may not be influenced by the overall lower levels of DNA methylation of the entire stage of development.

We then separated the methylation analysis into two sub-categories of read-outs: those associated with expressed (TPM≥1) or unexpressed (TPM<1) reference genes (Figure 6A, middle and right panels). The decreases in DNA methylation levels up-stream of the read-outs were slightly more pronounced and a minor methylation density difference of ~10% between expressed and non-expressed categories was observed. These differences in DNA methylation levels up-stream of different sub-categories of read-outs, however may not be necessary for their transcription initiation because both sub-categories were indeed transcribed.

For read-ins, a similar gradual yet more subdued decrease in DNA methylation up-stream of the transcribed regions, followed by a small increase or leveling off from the decrease in the transcribed read-in regions, were observed (Figure 6B, left panel). A sharp dip at the end of the transcribed read-in regions which was followed by a surge down-stream were observed. These regions in fact overlap with the TSS and promoter regions of the subsequent protein-coding genes. The changes therefore matched our previous data, which showed hypomethylated promoters and hypermethylated gene bodies of coding genes [10]. However, they were not expected to affect read-in transcription because these changes were down-stream of read-ins. We also separated the read-ins into two sub-categories of association with expressed or un-expressed reference genes (Figure 6B, middle and right panels). No major differences were observed in the DNA methylation between these two sub-categories of read-ins.

Finally, methylation in regions encompassing read-throughs did not show any distinct patterns in any stage studied. There was also no distinct stage-wide methylation density difference across different stages of development (Supplementary Figure 2). This could be caused by the combined factors of low numbers of read-throughs and even lower DNA methylation data at each stage of development for analysis input, resulting in many large variations in the final plots. Read-throughs, however, are products of bona fide termination failure. It is therefore reasonable to expect them to follow the methylation changes of the promoters of the up-stream reference genes. Some read-throughs were associated with reference genes that were not expressed, suggesting additional regulatory mechanisms beyond DNA methylation.

**Figure 7:**
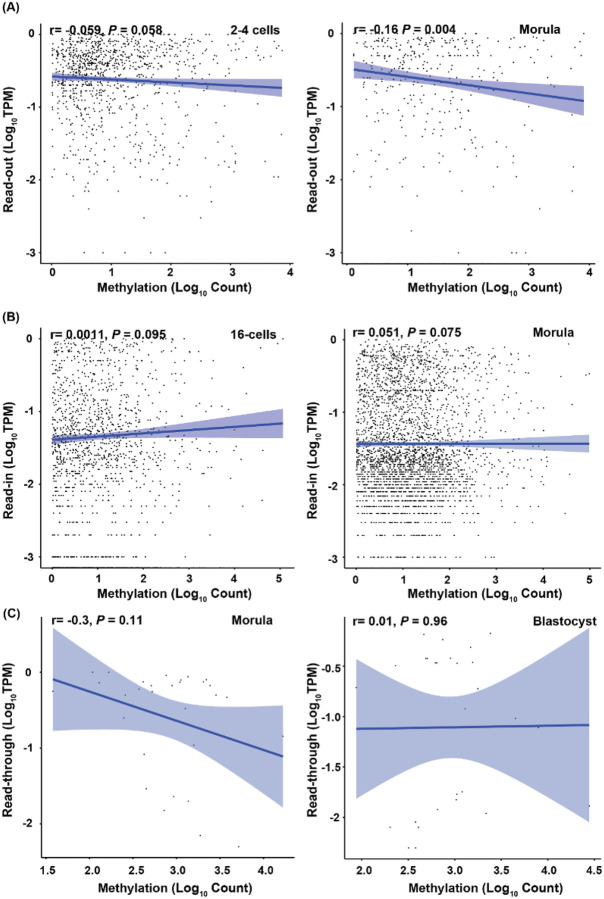
Pearson’s correlation between expression levels of read-outs (A), read-ins (B), or read-throughs (C) and their averaged methylation levels 1 kb up-stream of their TSS. Each dot in the plot represents the expression level of an intergenic transcript plotted against its averaged methylation counts. Only the highest and lowest correlations for each type of intergenic transcription are presented here. The rest can be found in Supplementary Figure S3.

To quantify the correlation of the levels between DNA methylation and intergenic transcription, we used Pearson’s correlation to analyze each specific intergenic transcript and its averaged DNA methylation counts 1 kb up-stream of the transcripts. If DNA methylation played a role in intergenic transcription, we would expect a significant negative correlation. Although relatively weak negative correlation was found for read-outs at morula (r= −0.16, *P*=0.004) and blastocyst (r= −0.15, *P*=0.00064) stages, these correlations were likely not biologically significant (Figure 7A, Supplementary Figure S3). The correlation for other intergenic transcriptions and stages were infinitesimal and insignificant (Figure 7B–C, Supplementary Figure S3).

### Transcription Factor Binding Motifs, and Polyadenylation Signals

Apart from read-throughs, which are resulted from bona fide transcription termination failures, the read-ins and read-outs could be potentially novel transcriptions of the intergenic regions. Altogether, we found 124, 125 and 11 different transcription factor binding motifs 300 bp up-stream of 8,439 read-outs, 11,882 read-ins and 1,095 read-throughs combined from all developmental stages. These motifs could potentially interact with 1,286, 1,514 and 145 transcription factors. Among all motifs, the most prevalent was TTTAAAAA, which was found in 23.2% of read-outs and could potentially bind seven transcription factors. TAAATAAA was found in 27.2% read-ins and could potentially bind 27 transcription factors. CAGCAGCAGCC was found in 9.9% read-throughs and could potentially bind 25 transcription factors. Interestingly, motifs for the transcription factor FOXK1 (Forkhead Box Protein K1), which has been associated with irregularity in transcription and seizure of embryo development in zebrafish [21], was found in all three categories of intergenic transcription in high abundance. Specifically, FOXK1 motif AGTCRTGTCCRACT was found in 13.1% of read-outs, AGTCGTGTCCRACTC in 14.8% read-ins, and GAGTCGGACACGACT in 6.8% read-throughs (Table 2). Other motifs that were present in read-outs and read-ins, could bind transcription factors important in embryonic development such as SOX2 (Supplementary Table 4). The motifs that could interact with the highest numbers of transcription factors in read-outs and read-ins were CCCCRCCCC, and CCCCACCCCC, which could interact with 63 and 72 transcription factors, respectively. Due to their similarity in sequences, these two motifs can interact with similar transcription factors including the Krüppel-like family of transcription factors (KLFs). The most interactive motif in read-throughs, AAACAAAACAAAMA, could bind 32 transcription factors including the Forkhead-box (FOX) gene family.

To determine if intergenic transcripts underwent RNA processing, we searched and found 7,367, 7,495 and 1,403 potential polyadenylation signals for the 8,439 read-outs, 11,882 read-ins and 1,094 read-throughs (combined from all developmental stages), respectively. The canonical polyadenylation sequence (AAUAAA) was present in 636 (8.2%), 792 (6.67%) and 129 (11.8%) read-outs, read-ins, and read-throughs whereas variant signal AAAAAA was the most prevalent in all three types of intergenic transcripts (read-out: 21%, read-in: 27.4%, and read-through: 35.5%). Many intergenic transcripts contained multiple copies of polyadenylation signals. These data provide strong evidence that intergenic transcripts were polyadenylated (Table 3).

## Discussion

Transcription termination is important in the regulation of gene expression [22, 23]. Many prior studies focused on termination failures and the generation of chimeric transcripts, which contained only transcripts termed here and in ARTDeco as read-throughs [4, 6, 24]. However, Roth et al. (2020) believed that read-throughs were not the only type of intergenic transcription resulted from termination failure. They expanded their definition to three categories of termination failure using transcription locations and close proximity to reference genes, which we analyzed here and termed read-outs, read-ins and read-throughs [8]. Roth et al. (2020) defined read-outs and read-ins as within 5 or 1 kb of annotated genes, and therefore, possibly a subset of lincRNA, rather than continuously transcribed chimeric RNAs. Utilizing ARTDeco, we identified naturally occurring intergenic transcripts during pre-implantation development and found them to be highly abundant in *in vivo* derived bovine oocytes and embryos. To our knowledge, this is the first report of transcription termination failure induced intergenic transcription using naturally occurring biological samples without *in vitro* culture.

Most previous analysis only characterized transcription termination failure or read-throughs. Our analysis, however, showed that read-outs and read-ins were much more abundant than read-throughs. On average, 30–60% of expressed genes were associated with read-outs or read-ins while for read-through, this is only about 10%. Interestingly, both read-outs and read-ins were less abundant in MII oocytes than embryos (Fig. 2B). This could be because MII oocytes have relatively or completely condensed chromatin which could be unconducive for genome-wide transcription [25]. Our analysis also showed intergenic transcripts were less expressed, accounting for 1–13.76% of total read counts of expressed genes (Supplementary Table 1A). This is in agreement with prior findings by Ulitsky & Bartel (2013) which described noncoding RNA to be around 10% of total gene expression [26].

Previously, thousands of differentially expressed genes were found between consecutive stages of *in vivo* [18] and in vitro bovine embryos [20]. These differences were especially pronounced between before and after EGA. Here we only identified hundreds of intergenic transcript differentials across a limited number of comparisons. This suggests that most differentially expressed genes were not accompanied with differentially expressed intergenic transcripts, thus further implying that there were likely specific regulations on intergenic transcription by genomic or epigenetic features. However, our analysis suggests that the regional DNA methylation patterns or up-stream methylation density were not likely among them. The lack of association of DNA methylation and intergenic transcription could be complicated by the fact that DNA methylation data from embryos had low coverage. Additionally, ARTDeco does not determine the exact TSS for intergenic transcripts, which could also have masked any methylation trend due to minor misalignments. Altogether, these questions should be re-visited once the methylation data for embryos are vastly present.

It is believed that termination failure induced intergenic transcription is the continuation of an mRNA of an expressed gene. To validate the hypothesis that intergenic transcription studied here, especially read-outs and read-ins, could also be resulted from novel transcription initiation, we searched and found a total of 260 motifs up-stream of intergenic transcription sites which can interact with 2,954 transcription factors. While diverse, some transcription factors were related to embryonic development. For example, Fox transcription factors have roles in development of embryo and differentiation [21, 27]. The transcription factor families of Zinc finger-Cys2His2, HMG (High mobility group), and MYB (myeloblastosis) [28] have all been shown to be actively dominant in early embryogenesis. Other transcription factors such as KLFs, which contains the zinc finger motif, and Sox2 which contains HMG domain, also play roles in differentiation [29] and embryogenesis [30], respectively. Additionally, at all stages of development, we found intergenic transcripts that were associated with reference genes that were un-expressed. For example, at the blastocyst stages, 3,582 read-outs, 4,168 read-ins and 411 read-throughs (Supplementary Table 1B) were expressed in the absence of reference gene expression. This strengthens our hypothesis that the intergenic transcription identified in early embryos includes novel transcription.

It was previously reported that lncRNA and lincRNA may be capped, spliced, and polyadenylated, resembling the processing of mRNAs [31, 32]. Similarly, we found abundant potential polyadenylation signals 100 bp up-stream of TES of intergenic transcripts studied here. These findings are logical because our RNA-seq datasets were generated from poly-A selected RNAs, suggesting that the intergenic transcripts we reported here were indeed polyadenylated. The findings of potential transcription factor binding motifs and polyadenylation signals strengthen our hypothesis that intergenic transcripts studied here are not simple continuation of mRNA transcription. Rather, they may be novel initiations and well-regulated.

## Conclusion

In conclusion, we found 1) widespread, naturally occurring intergenic transcription in all pre-implantation stages investigated, 2) the bovine genome has both “hot” and “cold” spots of intergenic transcription and some changed in correspondence to development, 3) while the expression levels of intergenic transcripts were lower than their reference genes, many were expressed in the absence of reference gene expression, 4) intergenic transcription does not seem to be related to the overall DNA methylation profiles 10 kb up-and down-stream of intergenic regions or correlated to the levels of DNA methylation 1 kb up-stream, 5) high numbers of transcription factor binding motifs were found up-stream of intergenic transcribed regions suggestion novel transcription initiation, 6) polyadenylation signals were present in high numbers of intergenically transcribed regions, implicating RNA processing. Together, intergenic transcription may be regulated by specific genome features and epigenetic mechanisms that are not related to those for their corresponding reference genes.

## Methods

### Datasets, Mapping, and Definitions

Data of RNA sequencing (GSE59186 [18]) and DNA methylation (GSE110400 [10] and GSE121758 [17]) that we generated previously using bovine *in vivo* developed oocytes and preimplantation embryos were used in this study. In the prior three publications, the data were used in the analysis of expressed genes and their correlation with DNA methylation, while in the present study, the data were used for intergenic expression analysis. The RNA-seq datasets contain two replicates of each stage of development (based on the stage coding of the International Embryo Technology Society): matured oocytes (MII), and embryos at 2-, 4-, 8-, 16-cell, early morula, compact morula, and blastocyst stages. The methylation datasets contain a total of nine replicates of MII stage, seven replicates of 2-cell stage, eight replicates of 4-cell stage, seven replicates of 8-cells, and three replicates each for 16-cell, early morula, compact morula and blastocyst stages. The total number of oocytes and embryos included were 59. Data analyses were initially performed with all stages separately. Results from the 2- and 4-cell stages were indifferent and therefore combined and referred to as 2–4 cells. Similarly, early and compact morulae were also combined and referred to as morula.

For mapping, RNA sequencing adapters were trimmed and read quality was filtered using TrimGalore (https://www.bioinformatics.babraham.ac.uk/projects/trim_galore/). Filtered reads were mapped to ARS-UCD1.2 (Bos Tau9) assembly using STAR [33]. The binary alignment map (BAM) files generated from the STAR aligner were processed through ARTDeco [8] to generate read counts and genome coordinates for expressed genes, and intergenic transcripts. Read counts were normalized to transcripts per million (TPM). Only expressed transcription with TPM ≥ 1 was taken into consideration for further analysis. The three types of intergenic transcripts were defined as follows, with TES and TSS referring to those of the expressed genes associated with intergenic transcripts unless noted otherwise. Read-outs were transcripts detected 5 kb after TES (to avoid detection of transcription that normally occurs in the region immediately 3′ of the polyadenylation signal-dependent cleavage site) and were therefore potentially new intergenic transcription initiation rather than termination failure (Fig. 1A). Read-ins were detected up-stream of genes, with detection starting from 1 kb up-stream of the reference gene’s TSS to avoid variations in TSS locations (Fig. 1B). The minimum and maximum lengths of read-outs and read-ins were 100 bp up to 15 kb. However, if another gene was present in the same locus of read-out or read-in, ARTDeco trimmed the length of read-out or read-in to one-third of the distance to that of the reference gene. Read-throughs were intergenic transcripts from TES, 4–15 kb in length and detected using rolling windows of 500 bp to ensure continuous transcription and therefore bona fide transcription termination failures (Fig. 1C). If a read-through extended into another down-stream gene, the down-stream transcription would be regarded as part of the read-through. ARTDeco identified read-outs before read-throughs and read-ins to avoid double accounting of intergenic transcription. In cases of overlaps of reference genes, too close in proximity (< 10 kb distance between gene ends), or one reference gene fell within the body of another reference gene (as is the case with many small RNAs), the read-out or read-in regions would be removed from consideration.

### Expression Levels, Visualization of Intergenic Expression, Principal Component Analysis and Gene Ontology

The numbers of expressed genes, read-outs, read-ins, and read-throughs were calculated based on developmental stages and individual chromosomes using the cutoff of TPM ≥ 1. Length distribution and 3D-principal component analysis (PCA) from TPMs of intergenic transcripts were plotted in R. To visualize genome-wide intergenic transcription, normalized bigwig files were generated from the BAM files using bedtools and plotted in integrated genome browser [34]. To determine if intergenic transcripts could be commonly expressed among different stages, Venn diagrams were generated among all stages (MII, 2–4 cell, 8-cell, 16-cell, morula and blastocyst), and before and after embryonic genome activation (EGA, between 4- to 8-cell stages for *in vivo* embryos [18] using the online tool InteractiVenn [35]. Gene ontology (GO) terms for common transcripts were identified using the web-based g: Profiler.

### Differential Expression of Intergenic Transcripts, Expression Comparison and Correlation to Reference Genes

Differential expression (DE) of read-outs, read-ins and read-throughs were identified using DESeq2 [36] between consecutive stages of pre-implantation development. Expression was considered differential if *P*-adjusted (*P*-adj) < 0.05 and log_2_ fold change (FC) ≥ 2. However, most comparisons did not generate many DEs. Therefore, the DEs from only the following stages were presented: MII vs. 8-cell, MII vs. blastocyst, 2–4 cell vs. blastocyst, and 8-cell vs. blastocyst.

To compare the levels of intergenic expression to the expression of their corresponding reference genes, ratios of read-outs, read-ins and read-throughs over expression of their corresponding reference genes were calculated and transformed into log_2_ scales in R. To determine if intergenic transcription was correlated to the expression of their corresponding reference genes, log_10_ transformed ratios of TPMs of read-outs, read-ins or read-throughs over their reference genes were subjected to Pearson’s correlation analysis in R. Results were considered significant if *P* < 0.05.

### Correlation of DNA Methylation to Intergenic Transcription, Detection of Potential Transcription Factor Binding Motifs and Polyadenylation Signals

Data of reduced representation bisulfite sequencing (RRBS, GSE110400) and whole genome bisulfite sequencing (WGBS, GSE121758) were combined and uplifted to the current genome version (ARS-UCD1.2) using the LiftOver tool [37]. DNA methylation profiles from replicates were combined using bedtools and CpG with read counts less than five were removed, consistent with our previous studies [17]. To visualize the overall DNA methylation patterns surrounding the intergenically transcribed regions, we used the coordinates of read-outs, read-ins, and read-throughs, and characterized regions 10 kb up- and down-stream of each intergenic transcript. The 20 kb + spans, including the intergenically transcribed regions, were separated into 300 bins (Fig. 1D). While the up- and down-stream regions were equally binned (100 bp per bin with a total of 100 bins), the number of bins for each intergenically transcribed region depended on its lengths (1 bin = length of intergenic transcript in bp/100 bp) as shown in Fig. 1D. In the case of the shortest and longest intergenic transcripts, 100 bp and 15 kb, the number of bp per bin would be 1 and 150, respectively. The average methylation level for each bin was calculated and density plot was generated in R.

To correlate the expression of intergenic transcripts (TPM ≥ 1) and DNA methylation levels, Pearson’s correlation was conducted using averaged DNA methylation of 1 kb up-stream of the TSS of expressed intergenic transcripts. Results were considered significant if *P* < 0.05.

To determine if intergenic transcripts might be new transcription initiation, we searched for transcription factor binding sites within 300 bp up-stream of TSS for intergenic transcripts, as it has been previously described that enhancer motifs are found within 300 bp up-stream of TSS for genes [38–40]. Intergenic transcripts of each category from all developmental stages were pooled, and transcription factor binding motif discovery was conducted using STREME [41]. Potential transcription factors that could interact with these motifs were identified using TOMTOM [42].

Our RNA-seq data were generated from poly-A selected RNAs. The fact that we were able to identify intergenic transcripts suggested that they might also be polyadenylated. We therefore searched for polyadenylation signals using pooled data of all developmental stages. The canonical polyadenylation signal sequence (AAUAAA) and 11 variants (AAAUAA, AUAAAA, AUUAAA, AUAAAU, AUAAAG, CAAUAA, UAAUAA, AUAAAC, AAAAUA, AAAAAA, AAAAAG) were searched 100 bp up-stream from the TES of intergenic transcripts using R.

## Figures and Tables

**Table 1:** Differentially expressed (DE) read-outs, read-ins and read-throughs between selected stages of development (*P*-value < 0.05, Log_2_FC ≥ 2).

Transcripts	Comparisons	Up-regulated	Down-regulated	Total
	MII vs. 8-cells	3	0	3
Read-out	MII vs. blastocyst	41	124	165
	2–4 cells vs. blastocyst	227	324	551
	8-cells vs. blastocyst	1	5	6
	MII vs. 8-cells	12	13	25
Read-in	MII vs. blastocyst	52	96	148
	2–4 cells vs. blastocyst	359	578	937
	8-cells vs. blastocyst	1	4	5
	MII vs. 8-cells	220	223	443
Read-through	MII vs. blastocyst	270	260	530
	2–4 cells vs. blastocyst	263	246	509
	8-cells vs. blastocyst	43	44	87

**Table 2:** Transcription factor binding motifs identified 300 bp up-stream of the TSS of all intergenic transcripts of each category combined for all developmental stages. Only abundant motifs are shown (for read-outs and read-ins, motifs that are present in more than 10% of intergenic transcripts are shown, whereas for read-throughs it was 5%).

Intergenic transcripts	Sequences of Motifs	No. (%) of sequence containing motif	No. of interactive transcription factors
	TTTAAAAA	1,951 (23.2%)	7
	AGTCRTGTCCRACT	1,101 (13.1%)	4
**Read-out**	CAGTCCATGGGRT	1,229 (14.6%)	3
**(8,439)**	AGCCCRCCAGGCTYC	965 (11.5%)	6
	ACTCCAGTAYTCTTG	1,027 (12.2%)	1
	GAGAAGGAAATGGCA	884 (10.5%)	12
	TAAATAAA	3,227 (27.2%)	27
	AGTCGTGTCCRACTC	1,762 (14.8%)	5
	CCCATGGACTGYAG	1,630 (13.7%)	8
**Read-in**	TATTTTWAA	1,475 (12.4%)	4
**(11,882)**	AGCGACTKAAC	1,327 (11.2%)	7
	GAGAAGGRAATGGCA	1,324 (11.1%)	12
	CACTCCAGTAYTCT	1,231 (10.4%)	1
	CCAGGCTYCYCTGTC	1,227 (10.3%)	10
	CAGCAGCAGCC	108 (9.9%)	25
	GGAGRAGGRRAYGG	102 (9.3%)	25
	GAGTCGGACACGACT	74 (6.8%)	4
**Read-through**	AATCCCATGGACDG	70 (6.4%)	7
**(1,094)**	ATTTTTAAAGA	67 (6.1%)	4
	ACCCACTCCAG	59 (5.4%)	14
	AAACAAAACAAAMA	59 (5.4%)	32
	CCAGGCAAGAATA	57 (5.2%)	6

**Table 3:** The numbers and percentages of polyadenylation signals found 100 bp up-stream of the TES of all intergenic transcripts combined from all developmental stages. Many intergenic transcripts contained more than one polyadenylation signal.

	Read-out (8,439)		Read-in (11,882)		Read-through (1,094)	
Poly-A Signals	No. (%) containing signal	Total signals detected	No. (%) containing signal	Total signals detected	No. (%) containing signal	Total signals detected
AAUAAA	636 (7.54%)	753	792 (6.67%)	894	129 (11.78%)	146
AAAAAA	634 (7.51%)	1,545	804 (6.77%)	2,053	122 (11.14%)	388
AAAAAG	650 (7.70%)	694	815 (6.86%)	880	98 (8.95%)	109
AAAAUA	688 (8.15%)	761	860 (7.24%)	983	133 (12.15%)	146
AAAUAA	636 (7.54%)	747	814 (6.85%)	950	120 (10.96%)	131
AUAAAA	559 (6.62%)	614	711 (5.98%)	784	113 (10.32%)	126
AUAAAC	289 (3.42%)	292	309 (2.60%)	315	45 (4.11%)	45
AUAAAG	353 (4.18%)	360	456 (3.84%)	466	58 (5.30%)	58
AUAAAT	419 (4.97%)	482	542 (4.56%)	583	63 (5.75%)	66
AUTAAA	469 (5.56%)	510	648 (5.45%)	687	90 (8.33%)	96
CAAUAA	292 (3.46%)	303	352 (2.96%)	362	45 (4.11%)	46
UAAUAA	288 (3.41%)	306	392 (3.30%)	439	44 (4.02%)	46
**Total**		**7,367**		**7,495**		**1,403**

## Data Availability

The RNA sequencing datasets analyzed in the current study was obtained from Gene Expression Omnibus (GEO) (www.ncbi.nlm.nih.gov/geo) under the accession number GSE59186 [18]. Similarly, dataset for methylation profiles were also obtained from Gene Expression Omnibus (GEO) under accession number GSE110400 [10] and GSE121758 [17].
